# Cross-Sectional Survey on the Current Role of Clinical Pharmacists among Antimicrobial Stewardship Programmes in Catalonia: Much Ado about Nothing

**DOI:** 10.3390/antibiotics12040717

**Published:** 2023-04-06

**Authors:** Daniel Echeverria-Esnal, Sergi Hernández, Anna Murgadella-Sancho, Ramón García-Paricio, Sara Ortonobes, Melisa Barrantes-González, Ariadna Padullés, Alexander Almendral, Montse Tuset, Enric Limón, Santiago Grau, on behalf of the Catalan Infection Control Antimicrobial Stewardship Programme (VINCat-ASP)

**Affiliations:** 1 Pharmacy Department, Hospital del Mar, Passeig Maritim 25-29, 08003 Barcelona, Spain; 2 Infectious Pathology and Antimicrobials Research Group (IPAR), Institut Hospital del Mar d’Investigacions Mèdiques (IMIM), Dr. Aiguader 88, 08003 Barcelona, Spain; 3 VINCat Programme Surveillance of Healthcare Related Infections in Catalonia, 08907 Barcelona, Spain; 4 Pharmacy Department, Hospital Moisès Broggi, Consorci Sanitari Integral (CSI), C/Oriol Martorell, 12, 08970 Sant Joan Despi, Spain; 5 Pharmacy Department, Hospital Municipal Badalona, Badalona Serveis Assistencials, 08911 Badalona, Spain; 6 Pharmacy Department, Consorci Corporació Sanitària Parc Taulí, Universitat Autònoma de Barcelona, 08208 Sabadell, Spain; 7 Pharmacy Department, Clinic Barcelona, 08036 Barcelona, Spain; 8 Department of Pharmacy, Hospital Universitari de Bellvitge-IDIBELL, L’Hospitalet de Llobregat, 08907 Barcelona, Spain; 9 Centro de Investigación Biomédica en Red de Enfermedades Infecciosas (CIBERINFEC), Instituto de Salud Carlos III, 28029 Madrid, Spain; 10 Department of Public Health, Mental Health and Mother-INFANT Nursing, School of Nursing, Faculty of Medicine and Health Sciences, University of Barcelona, 08007 Barcelona, Spain; 11 Department of Medicine, Universitat Pompeu Fabra, 08002 Barcelona, Spain

**Keywords:** antimicrobial stewardship, pharmacy, prospective audit and feedback, defined daily doses, days of treatment, information technology

## Abstract

Background. Antimicrobial resistance killed 1.27 million people in 2019, so urgent actions are desperately needed. Antimicrobial stewardship programmes (ASPs) are essential to optimize antimicrobial use. The objective was to acknowledge the current role of clinical pharmacists engaged in ASP activities in Catalonia. Methods. This was a cross-sectional survey shared through the Catalan Infection Control Programme (VINCat). The survey consisted of four sections and was sent by e-mail. Results. A total of 69.0% of the centres answered. Pharmacists dedicated a median of 5.0 h per week (2.1 h/week/100 acute care beds), representing 0.15 full time equivalents. The ASP lacked information technology (IT) support, as only 16.3% of centres automatically calculated defined daily doses and days of therapy. Those with less than 15% of their time available for ASPs conducted fewer clinical activities, especially prospective audits and feedback. Those without official infectious diseases training also performed fewer clinical activities, but training was less determinant than IT support or time. Pharmacists performed interventions mostly through annotation in the medical records. Conclusions. Clinical pharmacists from Catalonia dedicated to ASPs present an important lack of time and IT support to perform clinical activities. Pharmacists should also improve their clinical skills and try to conduct clinical advice to prescribers, either by phone or face-to-face.

## 1. Introduction

Antimicrobial resistance is a major problem currently, posing a growing threat to human health [1]. During the year 2019, 1.27 million people died because of antimicrobial resistance, so urgent solutions are desperately needed [1]. One of the main drivers of resistance is the overuse of antimicrobials, with studies showing that up to 50% of hospital prescriptions are unnecessary or inappropriate [2,3]. 

In this setting, antimicrobial stewardship programmes (ASPs) are essential [4]. According to the WHO, ASPs consist of a coherent set of actions which promote the appropriate use of antimicrobials, including actions at national and global levels [5]. Their goal is to improve antimicrobial use, and therefore patient outcomes [3]. They optimize clinical outcomes by increasing infection cure rates, reducing hospital length of stay, and even mortality through the optimization and reduction of their use, especially in intensive care units (ICUs) [3,6,7,8]. Furthermore, they reduce the unintended consequences of the use of these drugs, including the risk of antimicrobial-related adverse events, and selection and dissemination of antimicrobial resistant microorganisms, such as *Clostridioides difficile* infections [6,7,9]. Finally, they guarantee the use of cost-effective treatments [7,8].

Infectious disease (ID) physicians, clinical microbiologists, ICU physicians, pharmacists, and preventive medicine physicians and nurses, among others, form ASPs. In the hospital setting, clinical pharmacists exert an indispensable role in ASPs [6,10,11]. Although it is recommended that pharmacists have specific training in IDs [6,10,12], many hospitals have included clinical pharmacists without this training [6,13]. 

Despite some experiences showing the success of ASPs in Spain [2,4,8,14,15], the role, dedicated time, and type of interventions performed by clinical pharmacists have never been studied. A recent study published in the USA described the activities of clinical pharmacists engaged in ASPs, comparing the impact of these programmes on those with specific training and those who lack it [13]. Unfortunately, differences in healthcare systems hinder the applicability of these results to our clinical practice. 

The aim of this study was to analyse the results of a survey performed on clinical pharmacists involved in ASPs in Catalonia, to elucidate the current situation of the clinical pharmacy field in relation to ASPs.

## 2. Results

A total of 49/71 (69.0%) centres answered the survey, with a median of 189.0 (131.0–365.0) beds per centre. Main characteristics of hospitals and their computer tools are described in Table 1. 

As expected, statistically significant differences were found among groups in terms of hospital characteristics, as those from Group One presented a higher number of ICU beds and showed more complexity. 

**Figure 1:**
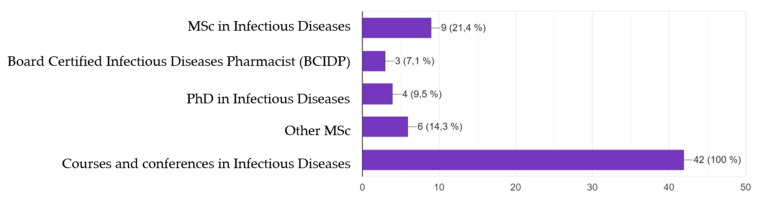
Clinical pharmacists’ training in infectious diseases. Participants may have more than one clinical training.

All hospitals included an ASP among their quality programmes that was supported by the senior leadership of the hospital (Table 1). ASPs were established 5.0 (4.0–8.0) years ago, with clinical pharmacists incorporated into the ASPs at that time. All centres include a minimum of one pharmacist. ASP pharmacists dedicated 5.0 (2.0–10.0) hours per week, and 2.1 (1.1–4.4) hours/week/100 acute care beds (ACBs). This represents 15.0% (6.5–26.0) of their available time, or 0.15 (0.1–0.3) full time equivalents (FTEs). Statistically significant differences were found among centres in terms of number of hours and FTEs. However, when corrected for 100 ACBs, those in Group One presented less time, though they did not achieve statistical significance. There were no differences in ID training, though pharmacists belonging to larger hospitals were better trained (Table 1 and Figure 1). Only 23 (46.9%) hospitals included information technology (IT) to perform ASP duties. Among those with IT, the available computer tools were limited, as only 28.6% had hard stop dates, 16.3% had automatic defined daily dose (DDD) and days of therapy (DOT) calculations, and 10.2% had automatic intravenous-to-oral switch recommendations. 

Table 2 shows the activities performed by clinical pharmacists, which are also depicted in the Supplementary Materials. They mainly participate in antimicrobial guidelines development, and provide recommendations on dosing and treatment duration, with >90% of pharmacists involved in these actions. All centres monitored antimicrobial consumption through DDD calculation. Among the 64.6% that performed therapeutic drug monitoring (TDM), the survey results reflect that 97.1% monitored vancomycin, 97.0% monitored aminoglycosides, 33.3% monitored triazoles, 24.2% monitored beta-lactams, and 15.2% monitored linezolid. 

Those in larger hospitals performed more investigation (although no differences were observed in the number of hours), teaching, and TDM. Despite not achieving statistical significance, clinical pharmacists from these hospitals performed fewer clinical activities than smaller hospitals. 

Table 3 shows the differences in performed clinical activities depending on the available time. Those pharmacists with more than 15% of their time dedicated to an ASP conducted more clinical activities. Remarkably, they conducted more revisions of restricted antimicrobials, prospective audit and feedback (PAF), antimicrobial spectrum and therapy duration recommendations, intravenous-to-oral switch recommendations, and monitoring antimicrobial-related side events. Furthermore, they provided more didactic education, investigation, and teaching. 

When the impact of ID training was analysed, Table 4 demonstrates that those with official training performed more interventions related to antimicrobial spectrum and monitoring of side events only.

**Figure 2:**
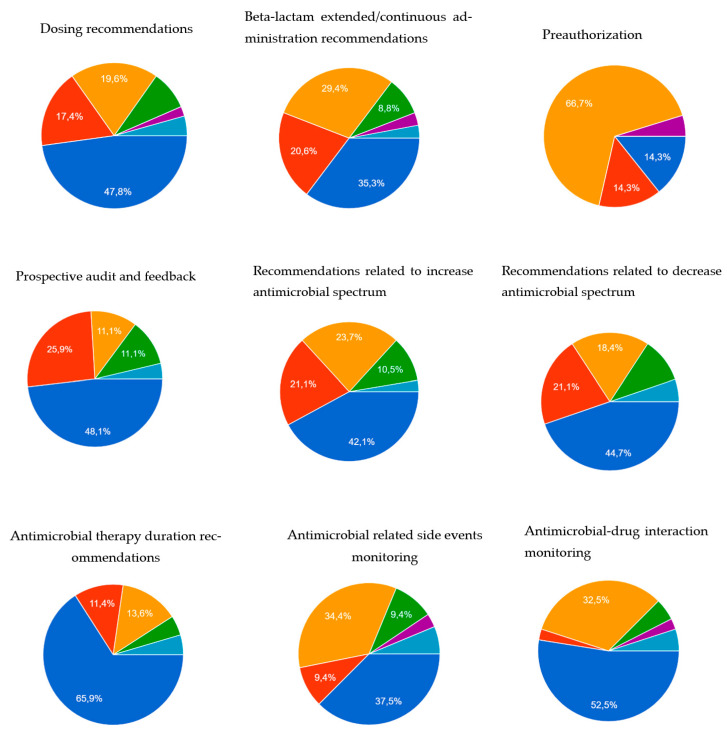

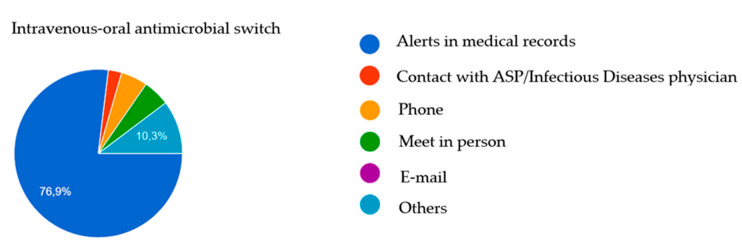
Methods of recommendation delivery of antimicrobial stewardship activities performed by clinical pharmacists.

Figure 2 details the methods used for recommendation delivery. Pharmacists mainly made their recommendations through medical records. Although, when the ASP action required the preauthorization of an antimicrobial prescription, pharmacists used the phone as the main recommendation delivery tool.

Table 5 describes the level of importance, time, and frequency required to perform ASP activities. Clinical pharmacists believed that that the most important activities were didactic education, guidelines development, and performance of antimicrobial spectrum-related recommendations. Likewise, didactic education, guidelines development, and investigation/teaching were the most time-consuming activities.

## 3. Discussion

There is a subjective perception that ASPs are being widely applied. However, in this regional survey we show important limitations of the current role of clinical pharmacists in relation to ASPs in Catalonia. One of the most striking findings of this study was the small number of hours dedicated to ASP activities, as well as the lack of IT support. Other relevant results include the small number of conducted clinical activities, especially in those hospitals with less available time, and the employed methods for recommendations delivery.

Clinical pharmacists had a surprisingly low number of hours dedicated to ASP activities, with only 5.0 h per week, 2.1 h/week/100 ACBs, or 0.15 FTEs. This is clearly less time compared to clinical pharmacists around the world, which show a mean of 18 h weekly (6.0 h in Africa, 32 h in North America) [16]. In Europe, an availability of 3.4–7.8 h/week/100 ACBs, or 0.75 FTEs per 100–400 beds, or 0.5 FTEs per 401–700 beds, has been observed [17,18]. Regarding different countries, recommendations for pharmacy staffing dedicated to ASPs vary, ranging from 0.25 FTE/100 ACBs in France to 1 FTE/100-300 ACBs, 1.2 FTE/300-500 ACBs, 2.0 FTE/501-1000 ACBs, and 3.0 FTE/ >1000 ACBs in the USA [13,19,20,21]. However, the USA health system has its particularities, and allocates more human resources to ASPs than other countries [16]. In Spain, the last standard published by PRAN (which is a strategic and action plan whose objective is to reduce the risk of selection and spread of resistance to antibiotics and, consequently, reduce the impact of this problem on the health of people and animals, sustainably preserving the efficacy of antibiotics) recommended the number of hours for the whole team (ID physicians, pharmacists, and microbiologists), depending on the level of the ASP. Staffing is a significant issue within ASPs, as, for example, a 0.5 increase in combined FTEs of ID physicians and ID pharmacists resulted in a 1.48-fold increase of ASP effectiveness [20]. In the specific case of pharmacists, each 0.5 increase in FTEs increased the odds of a programme being effective by 58% [20].

The wide ranges in staffing recommendations indicate the need to analyse, in depth, the variables that determine the real needs of hospitals. In this regard, other factors should be considered when staffing is discussed, beyond setting size: care complexity (ICU, transplant recipients), phase of ASP (initiation probably requires more resources compared to maintenance), and IT support [22]. 

ASPs from Catalonia presented fewer IT resources compared to other countries. The fact that only 16.8% of hospitals have an automatic tool to calculate DDD and DOT is alarming, as this reflects that in most of the pharmacies, this calculation is handmade. Although facing these adverse circumstances, it is noteworthy that pharmacy services of hospitals participating in the Catalan Infection Control Programme (VINCat) have been monitoring DDD in adults since 2008, and DOT in paediatrics since 2020. Furthermore, small hospitals do not have the need to include the diagnosis when prescribing, which makes ASP activities more difficult. These results contrast with other countries, such as the USA, France, or Italy, which show greater IT support for ASP activities [13,16,17,18,23]. The observed situation in Catalonia is worrisome. Lack of IT support is a significant barrier to perform stewardship interventions, as it could improve efficiency through myriad options, and help both physicians and pharmacists to implement more clinical and impactful interventions [24,25]. 

Clinical pharmacists in Catalonia performed fewer clinical activities compared to their counterparts in other areas of the world, especially in the case of PAF, antimicrobial spectrum-related recommendations, preauthorization, intravenous-to-oral switch, or collecting cultures and swabs [13,18]. One of the most alarming results was the difference in PAF, as those in Catalonia were (56.3%) carried out in a smaller proportion compared to those in the USA (82.2%), Australia (82.0%), or France (67%) [13,18]. This finding calls for reflection, as PAF is one of the most effective strategies, increasing the effectiveness of ASPs 3.82-fold [13,20].

Despite the lack of IT support, one of the main reasons for these findings could be time availability. Our study shows that those with more than 15% of weekly dedication time performed more high-value clinical activities, including PAF, revision of restricted antimicrobials, recommendations related to the antimicrobial spectrum, and to therapy duration. Another hypothesis could be differences in ID training, but according to our results, this fact was less determinant than the amount of dedicated time. Other reasons include that, likewise, ID physicians in our setting, especially in larger hospitals, mostly do PAF (19). 

Despite these differences in clinical activity, treatment duration recommendations were performed similarly. An explanation of this finding could be the 7-VINCut project initiated in 2019 within the VINCat programme. This project is based on a multidisciplinary intervention that attempts to reduce the duration of antimicrobial treatments in surgery wards [26]. This finding invites reflection, as it shows that conducting ASP interventions at an institutional level could alleviate burdens due to lack of IT support.

Most pharmacists rely upon alerts in electronic medical records to deliver their recommendations, probably because of a lack of time or wards rounding. While it is true that not every recommendation can be made verbally, pharmacists should ideally provide advice to prescribers, either by phone or face-to-face, and especially in clinical activities, such as PAF or spectrum-related recommendations [27].

Finally, another significant finding was the difference among pharmacists belonging to Group One and Two. Whereas those from Group One presented more ID training, IT resources, and conducted more TDM investigation and teaching, those in Group Two participated to a lesser extent in clinical activities. This is surprising, and may reflect the complexity of patients, as well as the fact that those are university hospitals, where the availability of ID physicians and medical specialists is greater than in hospitals from Groups Two and Three. Other hypotheses could be the less available time per ACB, as well as that in smaller hospitals, the contact is closer between the different providers.

This study is not without limitations. For instance, the interpretation of the survey’s questions could be different throughout all participants, leading to potential discrepancies in analysing the results. Moreover, the health system in Catalonia presents features that are difficult to extrapolate information to other countries or other regions in Spain. In this regard, participating centres are only those affiliated with the VINCat programme, so the results may not be fully applicable to other settings. Finally, given that we focused on pharmacists, we lack information regarding activities performed by other members of the ASP, as well as the proportion of ID physicians in each hospital. 

## 4. Materials and Methods

### 4.1. Study Design, Setting, and Participants

A cross-sectional online survey was shared through the VINCat programme [28]. VINCat is a programme of the Servei Català de la Salut that establishes a unified surveillance system for nosocomial infections in hospitals in Catalonia, and consists of a healthcare-associated infection surveillance programme involving seventy-one acute care hospitals in Catalonia (a region of Spain with 7.5 million inhabitants), including all public and some private centres, and ninety-seven long-term nursing homes. The VINCat programme includes several objectives. In this setting, the programme has implemented ASPs in hospitals, primary care, and long-term nursing homes (VINCat-ASP). The monitoring of annual antibiotic consumption, provided by the pharmacists of each centre, is one of the main indicators. All clinical pharmacists belonging to acute care hospitals affiliated with VINCat were invited to participate in the survey by e-mail. The invitation was sent on 22 July 2022, with reminders on 5 September and 14 September 2022. 

Hospitals within the VINCat programme are classified into three groups, depending on their size and complexity: Group One (more than five hundred beds), Group Two (200–499 beds), and Group Three (less than 200 beds). 

### 4.2. Survey Design

Two work meetings were held in May and June 2022 by the working party, constituted by VINCat ASP members. During the first meeting, we set the goals of the survey and prepared a draft, which was sent to investigators for review. During the second meeting, all discrepancies were resolved, and the final document was validated.

The survey was structured in four sections. The first section included questions related to hospital characteristics: number of beds, type of centre, and presence of special units or patients (ICU, paediatrics, solid organ/bone marrow transplant, onco-haematology, and others, such as cystic fibrosis or lung transplant). The second section asked about the ASP: presence of an institutionalized ASP, years since its implementation and the incorporation of the pharmacist into the team, the number of pharmacists and their weekly dedication to ASP in hours, the percentage of the weekly schedule dedicated to ASP, and the availability of IT or computer tools. With these data, FTEs were calculated. In those centres with IT, the availability of specific options to facilitate ASP duties was asked: automatic intravenous-to-oral switch recommendations, hard stop dates when prescribing, clinical decision support systems, time outs at 48–72 h, requirement to include diagnosis at the time of prescription, and automatic DDD and DOT calculations. 

The third section included questions regarding ID training: Master’s degree (MSc), Board Certified ID Pharmacist title (BCIDP), PhD, and courses and conferences. Those holding a MSc in ID were considered qualified when organized by the Autonomous University of Barcelona (UAB), the Spanish Agency of Medicines and Medical Devices (AEMPS), the National Plan of Antibiotic Resistance (PRAN), the Spanish Society of ID and Clinical Microbiology (SEIMC), or the Spanish Society of Hospital Pharmacy (SEFH). Other MSc refer to those not related to these institutions. Courses and conferences in ID were considered if they were organized by official organizations, such as VINCat, SEIMC, SEFH, or AEMPS. 

In the fourth section, aspects related to the activities carried out by pharmacists within ASPs were evaluated: revision and preauthorization of restricted antimicrobials, recommendations (dosing, beta-lactam administration strategies, antimicrobial spectrum, treatment duration, intravenous-to-oral switch, collection of cultures and swabs), PAF, monitoring of side events and drug–drug interactions, therapeutic drug monitoring (TDM), including which antimicrobials, and if recommendations based on plasmatic levels were made by ASP pharmacists or others, didactic education to other healthcare professionals, antimicrobial desensitisations and the annual number made, antimicrobial guidelines development, investigation and amount of hours dedicated per month, and teaching to residents. The method of recommendation delivery was also gathered, including by phone, in person, alerts in the medical record, e-mail, contact with ASP/ID physician, or others. Finally, participants were asked to order the importance they assigned to ASP actions and the spent time in those activities. They were requested to categorize their activities from 1 (greatest) to 5 (least).

The survey was designed using the Google Forms^®^ platform, and is available at https://docs.google.com/forms/d/e/1FAIpQLSeWugqUvTMQH4b95inDbEJZKEEnGuYL4chx8G9XbpmtjFMWTw/viewform?usp=sf_link (accessed on 25 June 2022). This survey was anonymous and voluntary, as centres did not receive any financial compensation for their participation. The estimated duration of the survey was 5–10 min. 

### 4.3. Statistical Analysis

Categorical variables were expressed as percentages and analysed using Fisher’s exact test. Continuous variables were described as median (Q1–Q3) and analysed using Kruskal–Wallis’s test or the Mann–Whitney U-Test, when appropriate. Nonparametrical analysis was conducted due to the small sample size. Missing values were excluded from the analysis. 

Statistical analyses were conducted depending on the assigned group based on VINCat classification (Groups One, Two, and Three), as well as on the weekly schedule dedicated to ASP activities (more or less than 15%, as this was the median time found in our study), and on the type of training (official training, including MSc, PhD, BCIDP, and other MSc, versus courses and conferences). *p*-values < 0.05 were considered statistically significant. Analyses were performed using IBM SPSS Statistics v21. 

## 5. Conclusions

This cross-sectional survey reflects the considerable number of issues that could be optimized within the ASP framework in Catalonia. This study calls for action, in terms of pharmacist staffing, as it reflects a significant lack of available time and a need for increased human resources. Health centre management should be aware that they should plan the workloads of the pharmacy services, considering the hours needed to dedicate to the ASP. IT support is also an essential part of ASPs, and should be funded and developed to allow physicians and pharmacists to improve the quality of their interventions. The observed results also set the path for pharmacists to improve their ability to conduct clinical activities. Pharmacists should upgrade their ID training, agree with the rest of the ASP regarding which interventions should be prioritized, and improve the methods of communicating with medical staff, especially for prospective audits and feedback. The obtained results will allow the establishment of a roadmap to improve the quality of ASPs in Catalonia. 

## Figures and Tables

**Table 1:** Characteristics of hospitals and available computer tools of antimicrobial stewardship programmes.

	Total	Group 1 (≥500 Beds)	Group 2 (200–499 Beds)	Group 3 (<200 Beds)	*p* Value
Number of hospitals included	49	7	16	26	
Number of beds	189.0 (131.0–365.0)	723.0 (650–763)	309.0 (212.5–395.0)	134.0 (100.0–159.3)	**<0.001**
Teaching hospital	21 (42.9)	6 (85.7)	9 (56.3)	6 (23.1)	**0.005**
Private hospital	8 (16.3)	0 (0)	2 (12.5)	6 (23.1)	0.301
Intensive care unit *Number of beds*	32 (65.3) *12.0 (8.0–36.5)*	7 (100.0) *90.0 (38–117)*	15 (93.8) *12.0 (10–20)*	10 (38.5) *8.0 (5.5–9.3)*	**<0.001** **<0.001**
Solid organ or bone marrow transplantation units	9 (18.4)	5 (71.4)	1 (6.3)	3 (11.5)	**<0.001**
Onco-haematology units	35 (71.4)	6 (85.7)	12 (75.0)	17 (65.4)	0.531
Paediatric units	33 (67.3)	4 (57.1)	13 (81.3)	16 (61.5)	0.344
Others (cystic fibrosis, lung transplant)	6 (12.2)	3 (42.9)	2 (12.5)	1 (3.8)	**0.020**
*ASP characteristics*					
Number of years since ASP establishment	5.0 (4.0–8.0)	5.0 (3.0–7.0)	6.0 (4.3–10.0)	4.0 (3.0–6.5)	0.118
Number of years since clinical pharmacists were incorporated into ASP	5.0 (4.0–8.0)	5.0 (3.0–7.0)	6.0 (4.0–10.0)	4.0 (3.0–6.5)	0.364
Number of pharmacists in ASP	1.0 (1.0–1.0)	1 (1–2)	1 (1–1)	1 (1–1)	0.122
Number of hours dedicated by clinical pharmacists to ASP per week	5.0 (2.0–10.0)	10.0 (2.0–20)	7.0 (5.3–10.0)	3.0 (1.0–7.0)	**0.012**
Number of hours dedicated by clinical pharmacists to ASP per week/100 acute care beds	2.3 (1.3–4.9)	1.5 (0.3–2.5)	2.6 (1.7–4.5)	2.1 (1.2–6.3)	0.130
Full time equivalents	0.15 (0.1–0.3)	0.28 (0.1–0.5)	0.21 (0.2–0.3)	0.1 (0.04–0.2)	**0.003**
ID Training *MSc, PhD, BCIDP* *Courses and conferences*	42 *14 (28.6)* *28 (57.1)*	7 (100.0) *4 (57.1)* *3 (42.9)*	15 (93.8) *6 (40.0)* *9 (60.0)*	20/25 (80.0) *4 (20.0)* *16 (80.0)*	0.240 *0.158* *0.254*
*ASP computer tools*					
Computer tools to perform ASP	23 (46.9)	4 (57.1)	9 (56.3)	10 (38.5)	0.449
Computer tool includes a specific section of recommendation for intravenous-to-oral switch	5 (10.2)	2 (28.6)	0 (0)	3 (11.5)	0.108
Computer tool includes the need to prescribe the length of treatment at the time of prescription	14 (28.6)	3 (42.9)	5 (31.3)	6 (23.1)	0.565
Computer tool includes clinical decision support systems	2 (4.1)	1 (14.3)	1 (6.3)	0 (0)	0.206
Computer tool includes time outs at 48–72 h of antimicrobial prescribing	1 (2.0)	0 (0)	0 (0)	1 (3.8)	0.637
Computer tool includes the need to include the diagnosis at the time of prescription	7 (14.3)	2 (28.6)	5 (31.3)	0 (0)	**0.010**
Computer tool includes defined daily doses automatic calculation	8 (16.3)	3 (42.9)	3 (18.8)	2 (7.7)	0.078
Computer tool includes days of treatment automatic calculation	8 (16.3)	2 (28.6)	4 (25.0)	2 (8.0)	0.239

Categorical variables presented in percentages. Continuous variables presented in median (Q1–Q3). ASP: antimicrobial stewardship programme; ID: infectious disease; BCIDP: board certified infectious diseases pharmacist.

**Table 2:** Antimicrobial stewardship activities performed by clinical pharmacists according to the type of hospital.

	Total (*n* = 48)	Group 1 (*n* = 7)	Group 2 (*n* = 16)	Group 3 * (*n* = 25)	*p* Value
*ASP activities*					
Number of annual meetings	6.0 (5.0–8.0)	6.0 (5.0–12.0)	12.0 (6.0–40.0)	4.0 (4.0–31.5)	0.088
Revision of restricted antimicrobials	42 (87.5)	5 (71.4)	16 (100.0)	21 (84.0)	0.121
Dosing recommendations	46 (95.9)	7 (100.0)	16 (100.0)	23 (92.0)	0.383
Beta-lactam extended/continuous administration recommendations	34 (70.8)	4 (57.1)	14 (87.5)	16 (64.0)	0.187
Didactic education	26 (55.3)	5 (71.4)	10/15 (66.7)	11 (44.0)	0.245
Preauthorization	21 (43.8)	3 (42.9)	10 (62.5)	8 (32.0)	0.158
Prospective audit and feedback	28 (58.3)	4 (57.1)	13 (81.3)	11 (44.0)	0.062
Antimicrobial spectrum-related recommendations	38 (80.9)	6 (85.7)	14 (87.5)	18/24 (75.0)	0.579
Antimicrobial therapy duration recommendations	44 (91.7)	5 (71.4)	16 (100)	23 (92.0)	0.074
Antimicrobial-related side events monitoring	32 (68.1)	5 (71.4)	12 (75.0)	15 (62.5)	0.693
Antimicrobial–drug interaction monitoring	41 (85.4)	7 (100.0)	15 (93.8)	19 (76.0)	0.145
Antimicrobial desensitisation *Number of desensitisations*	12 (25.0) *5.0 (4.5–5.0)*	2 (28.6) *24.5 (0–49.0)*	6 (37.5) *4.0 (1.0–5.0)*	4 (16.0) *1.5 (1.0–15.5)*	0.292 *0.262*
Intravenous-to-oral antimicrobial switch	39 (81.3)	6 (85.7)	14 (87.5)	19 (76.0)	0.621
Antimicrobial guidelines development	47 (97.9)	7 (100.0)	16 (100.0)	24 (96.0)	0.625
Recommendations to perform cultures or swabs	16 (33.3)	1 (14.3)	9 (56.3)	6 (24.0)	0.052
Investigation *Number of hours in a month*	12 (25.0) *4.0 (5.0–27.5)*	4 (57.1) *4.0 (2.5–31.0)*	5 (31.3) *5.0 (3.0–30.0)*	3 (12.0) *2.0 (0–2.0)*	**0.040** *0.381*
Teaching	20 (41.7)	5 (71.4)	10 (62.5)	5 (20.0)	**0.006**
Therapeutic drug monitoring *Recommendations are made by ASP pharmacists*	34 (70.8) *18 (52.9)*	7 (100.0) *2 (28.6)*	13 (81.3) *10 (76.9)*	14 (56.0) *6 (42.9)*	**0.041** *0.073*

One hospital did not answer these questions. * Categorical variables presented in percentages. Continuous variables presented in median (Q1–Q3). ASP: antimicrobial stewardship programme.

**Table 3:** Type of performed antimicrobial stewardship activity depending on the weekly available time.

	≥15% of Time (*n* = 26)	<15% of Time (*n* = 22) *	*p* Value
Type of hospital			**0.005**
*Group 1* *Group 2* *Group 3*	*5 (19.2)* *13 (50.0)* *8 (30.8)*	*2 (9.1)* *3 (13.6)* *17 (77.3)*	
Number of annual meetings	11.0 (4.8–40.0)	5.0 (4.0–12.0)	0.124
Revision of restricted antimicrobials	26 (100.0)	16 (72.7)	**0.006**
Dosing recommendations	26 (100.0)	20 (90.9)	0.205
Beta-lactam extended/continuous administration recommendations	24 (92.3)	10 (45.5)	**<0.001**
Didactic education	19/25 (76.0)	7 (31.8)	**0.003**
Preauthorization	14 (53.8)	7 (31.8)	0.153
Prospective audit and feedback	21 (80.8)	7 (31.8)	**0.001**
Antimicrobial spectrum related recommendations	24/25 (96.0)	14 (63.6)	**0.008**
Antimicrobial therapy duration recommendations	26 (100.0)	18 (81.8)	**0.038**
Antimicrobial related side events monitoring	22 (84.6)	10/21 (47.6)	**0.011**
Antimicrobial–drug interaction monitoring	24 (92.3)	17 (77.3)	0.223
Antimicrobial desensitisation *Number of desensitisations*	7 (26.9) *4.0 (1.5–5.0)*	5 (22.7) *1.0 (1.0–34.5)*	1.000 *0.841*
Intravenous-to-oral antimicrobial switch	25 (96.2)	14 (63.6)	**0.007**
Antimicrobial guidelines development	26 (100.0)	21 (95.5)	0.458
Recommendations to perform cultures or swabs	11 (42.3)	5 (22.7)	0.221
Investigation *Number of hours in a month*	10 (38.5) *4.5 (3.0–17.5)*	2 (9.1) *1.0 (0–1.0)*	**0.023** ***0.030***
Teaching	16 (61.5)	4 (18.2)	**0.003**
Therapeutic drug monitoring *Recommendations are made by ASP pharmacists*	21 (80.8) *13 (61.9)*	13 (59.1) *5 (38.5)*	0.122 *0.291*

* One hospital did not answer questions related to activities in this group. Categorical variables presented in percentages. Continuous variables presented in median (Q1–Q3). ASP: antimicrobial stewardship programme.

**Table 4:** Type of performed antimicrobial stewardship activity depending on infectious disease training.

	MSc, BCIDP, PhD (*n* = 14)	Courses and Conferences (*n* = 28)	*p* Value
Type of hospital			0.158
*Group 1* *Group 2* *Group 3*	*4 (28.6)* *6 (42.9)* *4 (28.6)*	*3 (10.7)* *9 (32.1)* *16 (57.1)*	
Number of annual meetings	10.0 (4.5–40.0)	6.0 (4.0–36.0)	0.742
Revision of restricted antimicrobials	13 (92.9)	25 (89.3)	1.000
Dosing recommendations	14 (100.0)	27 (96.4)	1.000
Beta-lactam extended/continuous administration recommendations	12 (85.7)	19 (67.9)	0.283
Didactic education	10 (71.4)	15/27 (55.6)	0.501
Preauthorization	7 (50.0)	13 (46.4)	1.000
Prospective audit and feedback	10 (71.4)	15 (53.6)	0.331
Antimicrobial spectrum related recommendations	13/13 (100.0)	20 (71.4)	**0.040**
Antimicrobial therapy duration recommendations	13 (92.9)	26 (92.9)	1.000
Antimicrobial-related side events monitoring	13 (92.9)	17 (60.7)	**0.036**
Antimicrobial–drug interaction monitoring	14 (100.0)	24 (85.7)	0.283
Antimicrobial desensitisation *Number of desensitisations*	6 (42.9) *4.5 (2.5–38.0)*	6 (21.4) *1.0 (1.0–8.8)*	0.169 *0.171*
Intravenous-to-oral antimicrobial switch	13 (92.9)	22 (78.6)	0.392
Antimicrobial guidelines development	14 (100.0)	27 (96.4)	1.000
Recommendations to perform cultures or swabs	5 (35.7)	10 (35.7)	1.000
Investigation *Number of hours in a month*	5 (35.7) *8.0 (3.5–45.0)*	6 (21.4) *3.0 (1.5–6.3)*	0.459 *0.126*
Teaching	9 (64.3)	11 (39.3)	0.192
Therapeutic drug monitoring *Recommendations are made by ASP pharmacists*	11 (78.6) *5 (45.5)*	19 (67.9) *11 (57.9)*	0.485 *0.707*

Categorical variables presented in percentages. Continuous variables presented in median (Q1–Q3). MSc: Master’s degree; BCIDP: board certified infectious diseases pharmacist; ASP: antimicrobial stewardship programme.

**Table 5:** Level of importance, time, and frequency required to perform antimicrobial stewardship activities.

	1	2	3	4	5
Level of importance of performed ASP activities	Didactic education and guidelines development: 21.7 Antimicrobial spectrum-related recommendations: 13.0	Antimicrobial spectrum-related recommendations: 28.3 Antimicrobial duration-related recommendations: 19.6 Beta-lactam administration: 13.0	Antimicrobial duration: 37 Intravenous-to-oral switch: 15.2 Antimicrobial spectrum: 13.0	TDM: 17.4 Antimicrobial duration: 15.2 Guidelines development and antimicrobial spectrum: 13.0	Didactic education: 18.8 Antimicrobial spectrum: 16.7 Guidelines development: 14.6
Time required for ASP activities	Guidelines development: 25.6 Didactic education: 23.3 Investigation/teaching: 20.9	TDM: 18.2 Didactic education: 15.9 Guidelines development: 13.6	Guidelines development: 34.1 Beta-lactam administration, TDM, didactic education, side-events monitoring: 11.4	Didactic education: 22.7 TDM: 13.6 Antimicrobial spectrum: 11.4	Antimicrobial duration: 20.5 TDM: 13.6 Investigation/teaching, side-events monitoring: 11.4

Order of numbers: one (greatest), five (least). ASP: antimicrobial stewardship programme; TDM: therapeutic drug monitoring. Numbers expressed as percentage.

## Data Availability

Data are available upon request to the authors.

## Supplementary Materials

The following supporting information can be downloaded at: https://www.mdpi.com/article/10.3390/antibiotics12040717/s1, Figure S1: Antimicrobial stewardship activities performed by clinical pharmacists.
