# Supplementary material for: Cross-Sectional Survey on the Current Role of Clinical Pharmacists among Antimicrobial Stewardship Programmes in Catalonia: Much Ado about Nothing
Source: Antibiotics (Basel). 2023 Apr 6;12(4):717. doi: 10.3390/antibiotics12040717 (PMC10135239; doi:10.3390/antibiotics12040717)

**Figure S1.** Antimicrobial stewardship activities performed by clinical pharmacists.

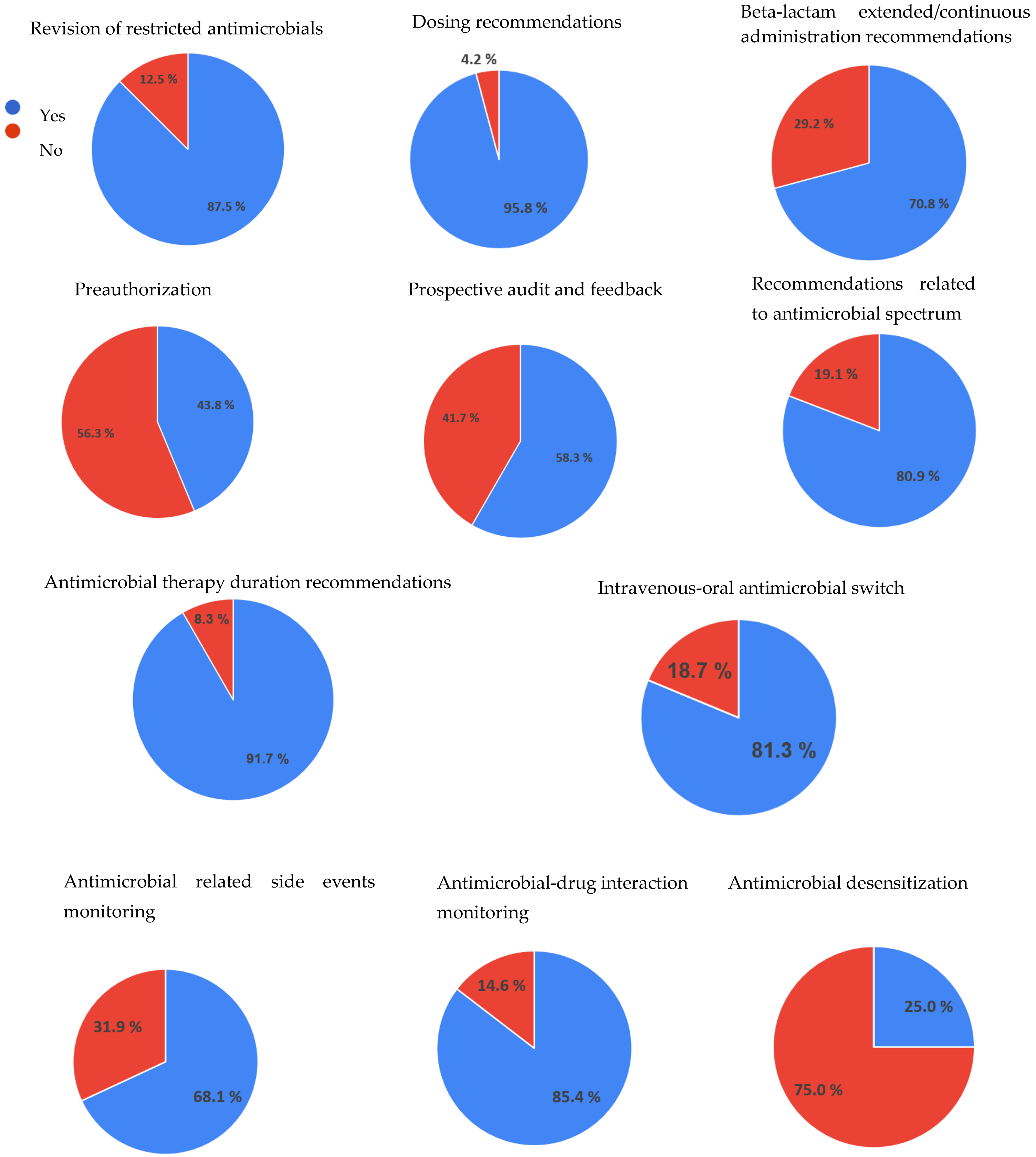

Recommendations to perform cultures or swabs

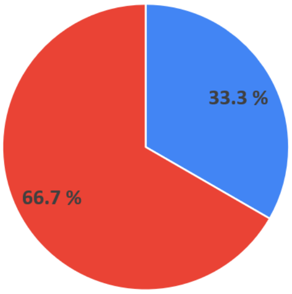


Investigation

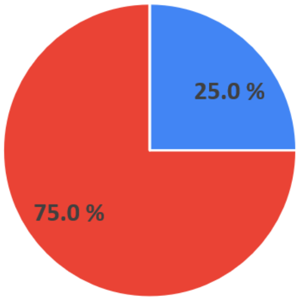


Therapeutic drug monitoring

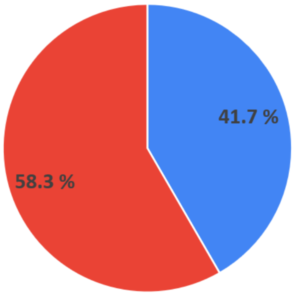

Supplement: Supplementary file 1 [file antibiotics-12-00717-s001.zip › antibiotics-2319442-supplementary.pdf]
